# A new efficient trial design for assessing reliability of ankle-brachial index measures by three different observer groups

**DOI:** 10.1186/1471-2261-6-33

**Published:** 2006-07-27

**Authors:** Heinz G Endres, Christian Hucke, Tim Holland-Letz, Hans-Joachim Trampisch

**Affiliations:** 1 Department of Medical Informatics, Biometry and Epidemiology, Ruhr-University, Bochum, D-44801 Bochum, Germany

## Abstract

**Background:**

The usual method of assessing the variability of a measure such as the ankle brachial index (ABI) as a function of different observer groups is to obtain repeated measurements. Because the number of possible observer-subject combinations is impractically large, only a few small studies on inter- and intraobserver variability of ABI measures have been carried out to date. The present study proposes a new and efficient study design. This paper describes the study methodology.

**Methods:**

Using a partially balanced incomplete block design, six angiologists, six primary-care physicians and six trained medical office assistants performed two ABI measurements each on six individuals from a group of 36 unselected subjects aged 65–70 years. Each test subject is measured by one observer from each of the three observer groups, and each observer measures exactly six of the 36 subjects in the group. Each possible combination of two observers occurs exactly once per patient and is not repeated on a second subject. The study involved four groups of 36 subjects (144), plus standbys.

**Results:**

The 192 volunteers present at the study day were similar in terms of demographic characteristics and vascular risk factors: mean age 68.6 ± 1.7; mean BMI 29.1 ± 4.6; mean waist-hip ratio 0.92 ± 0.09; active smokers 12%; hypertension 60.9%; hypercholesterolemia 53.4%; diabetic 17.2%. A complete set of ABI measurements (three observers performing two Doppler measurements each) was obtained from 108 subjects. From all other subjects at least one ABI measurement was obtained. The mean ABI was 1.08 (± 0.13), 15 (7.9%) volunteers had an ABI <0.9, and none had an ABI >1.4, i.e. a ratio that may be associated with increased stiffening of the arterial walls.

**Conclusion:**

This is the first large-scale study investigating the components of variability and thus reliability in ABI measurements. The advantage of the new study design introduced here is that only one sixth of the number of theoretically possible measurements is required to obtain information about measurement errors. Bland-Altman plots show that there are only small differences and no systematic bias between the observers from three occupational groups with different training backgrounds.

## Background

Coronary heart disease and stroke as manifestations of atherosclerosis are among the leading causes of death. Consequently, there has been an intensive search for measures of atherosclerosis burden that would indicate the cardiovascular and overall risk of the individual patient [1]. An early indicator of possible generalized atherosclerosis is peripheral arterial disease (PAD), which refers to the manifestation of atherosclerosis below the aortal bifurcation [2,3].

An early and reproducible diagnosis of PAD is important for identifying high-risk patients as early as possible. Only a minority of patients exhibit the symptoms of intermittent claudication. Data from the literature suggest that for every patient with intermittent claudication, there are at least three with asymptomatic PAD [4].

The introduction of Doppler sonography has made it possible for general physicians to diagnose asymptomatic PAD by determining the ankle-brachial index (ABI), which represents the ratio of ankle to brachial systolic blood pressure (SBP) [5,6]. ABI values below 0.9 are considered pathological (presence of PAD) [7,8]. Compared to angiography, the sensitivity of a low ABI for leg artery stenosis of ≥ 50% is about 90%, and the specificity is about 98% [9]. In a recent systematic review, the specificity of low ABI to predict future cardiovascular outcomes was high (e.g. 88% for cardiovascular mortality), but the sensitivity was low (41%) [10].

To establish ABI determination as a screening procedure for asymptomatic PAD, the interobserver variability (same subject, different observers) and intraobserver variability (same subject, same observer) of ABI measures must be known. Of particular interest is how these two types of variability differ depending on the observer's level of specialization. The present study therefore set out to determine inter- and intraobserver variability of ABI measurements by three groups of observers (angiologists, general physicians and medical office assistants) and with a sufficiently large number of randomly selected subjects, age 65 to 70, free of serious disease and without symptomatic PAD (the target group of ABI screening measurements). In order to avoid having to make very large numbers of measurements (impractical for both subjects and observers), a study design was developed that provides the necessary information with one sixth the number of Doppler measures that would otherwise have to be performed. The paper deals with the study methodology.

## Methods

### Study population

**Figure 1:**
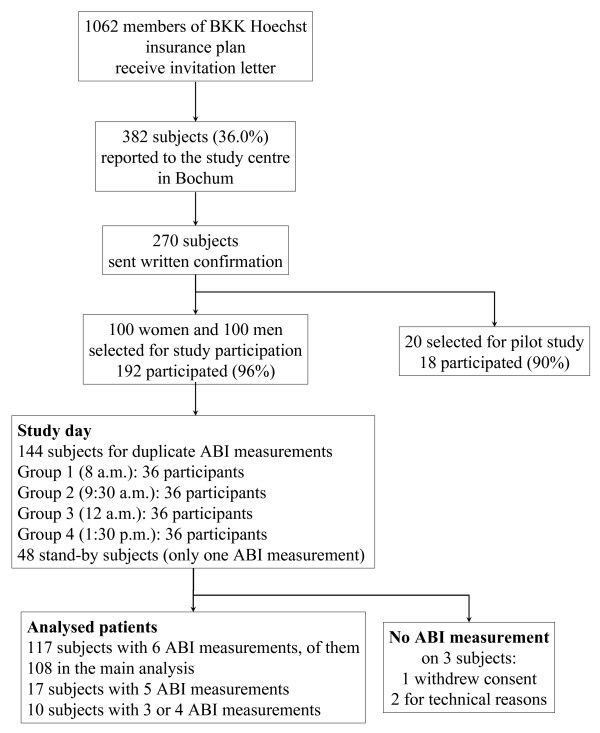
Patient disposition and study flow.

Volunteers were eligible for inclusion in the study if they were members of a public health insurance plan (in this case BKK Hoechst), between the ages of 65 and 70, resident in Frankfurt-Hoechst, ambulatory without wheelchair or walking aids, able to understand the study and able to provide written informed consent. Exclusion criteria were: need for nursing care of any kind, serious disease such as cancer, any amputation of upper or lower extremities, and a history of stroke with hemiplegia. Figure 1 shows the study flow. A total of 1062 subjects meeting the inclusion criteria received a letter from the BKK Hoechst insurance plan inviting them to participate in the study.

A total of 382 people (36.0%) responded to the study center in Bochum, of whom 310 were interested in participating and 270 signed the statement of informed consent. To ensure that at least 144 subjects would be available for the ABI measurements on the day of the study (four groups of 36), the first 100 men and the first 100 women to respond to the invitation were asked to attend. As a result there were 14 more participants per group than the minimum of 36 required (stand-by). The four groups were asked to arrive at 1.5- to 2.5-hour intervals. Another 20 subjects whose data were not to be included in the analysis of results were asked to participate in a "dress rehearsal" one day before the actual study day. The study was conducted in accordance with the Helsinki Declaration and the Guideline for Good Epidemiological Practice (GEP) issued by the German Working Group on Epidemiology [11]. The study was approved by the ethics committee of the Ruhr-University of Bochum.

### Observers

The duplicate ABI measurements were performed by six angiologists, six general physicians (GPs) and six medical office assistants with special training in Doppler measurements (MAs). The angiologists (internists with a specialization in angiology) were selected from the "centers of excellence" (coordinating and training centers) of the German Epidemiological Trial on Ankle-Brachial Index (getABI) [8,12]. The GPs were also investigators in getABI and were chosen by the angiologists. The MAs were employees of the angiologists and specialized in ABI measurements. The basic physical examinations were performed by separate observers (three angiologists and three GPs) who were not involved in performing the duplicate ABI measurements. These extra angiologists/GPs also performed a single ABI measurement on the stand-by subjects.

### Study design

On the study day (December 12, 2005), all volunteers first underwent a physical examination by an angiologist or GP. This basic examination covered weight and height for calculating BMI (seca 703 electronic column scale with built-in seca 220 height measuring rod, seca GmbH Hamburg, Germany), waist and hip circumference for calculating waist-hip ratio, and patient history regarding vascular-related comorbidities (Table 1), obtained in a standardized manner and recorded on case report forms.

**Table 1:** Demographic data for the 192 subjects who reported on day of study

	**Group 1**	**Group 2**	**Group 3**	**Group 4**	**Group 5 Stand-bys**	**Total**	p-values (global test of all 5 groups)*	**Validation groups (Groups 1, 2 and 3)**
**Number of participants**	**36**	**36**	**36**	**36**	**48**	**192**		**108**
**Age: mean (± SD)**	68.8 (1.4)	68.7 (1.5)	68.5 (1.5)	68.3 (1.5)	68.7 (2.4)	68.6 (1.7)	**0.619**	68.7 (1.5)
**Sex: N (%)**							**0.530**	
**M**	15 (41.7%)	20 (55.6%)	15 (41.7%)	19 (52.8%)	27 (56.3%)	96 (50%)		50 (46.3%)
**F**	21 (58.3%)	16 (44.4%)	21 (58.3%)	17 (47.2%)	21 (43.7%)	96 (50%)		58 (53.7%)
**BMI: mean (± SD)**	28.5 (4.3)	29.2 (4.7)	29.2 (4.0)	29.0 (5.2)	29.3 (4.9)	29.1 (4.6)	**0.956**	29.0 (4.3)
**< 25: N (%)**	5 (13.9%)	7 (19.4%)	2 (5.6%)	5 (13.9%)	11 (22.9%)	30 (15.6%)	**0.455**	14 (13.0%)
**25 – < 30: N (%)**	19 (52.8%)	16 (44.4%)	19 (52.8%)	18 (50.0%)	16 (33.3%)	88 (45.8%)		54 (50.0%)
**>= 30: N (%)**	12 (33.3%)	13 (36.1%)	15 (41.7%)	13 (36.1%)	21 (43.8%)	74 (38.5%)		40 (37.0%)
**WHR: mean (± SD)**	0.91 (0.09)	0.93 (0.09)	0.91 (0.08)	0.95 (0.09)	0.92 (0.08)	0.92 (0.09)	**0.408**	0.92 (0.09)
**M**	0.97 (0.05)	1.0 (0.04)	0.99 (0.07)	1.0 (0.06)	0.97 (0.07)			0.99 (0.05)
**F**	0.86 (0.09)	0.84 (0.05)	0.86 (0.05)	0.89 (0.07)	0.87 (0.05)			0.86 (0.06)
**Smoker status: N (%)**								
**Active smoker**	3 (8.3%)	3 (8.3%)	4 (11.1%)	7 (19.4%)	6 (12.5%)	23 (12.0%)	**0.538**	10 (9.3%)
**Former smoker**	17 (47.2%)	17 (47.2%)	10 (27.8%)	11 (30.6%)	19 (39.6%)	74 (38.5%)		44.(40.7%)
**Non-smoker**	16 (44.4%)	16 (44.4%)	22 (61.1%)	18 (50.0%)	23 (47.9%)	95 (49.5%)		54 (50.0%)
**Hypertension: N (%)**								
**Yes**	20 (57.1%)	20 (57.1%)	21 (60.0%)	20 (55.6%)	34 (70.8%)	115 (60.8%)	**0.571**	61 (58.1%)
**No**	15 (42.9%)	15 (42.9%)	14 (40.0%)	16 (44.4%)	14 (29.2%)	74 (39.2%)		44 (41.9%)
**Missing**	1	1	1	0	0	3		3
**Hypercholesterolemia: N (%)**								
**Yes**	18 (52.9%)	16 (45.7%)	23 (65.7%)	16 (55.2%)	21 (48.8%)	94 (53.4%)	**0.501**	57 (54.8%)
**No**	16 (47.1%)	19 (54.3%)	12 (34.3%)	13 (44.8%)	22 (51.2%)	82 (46.6%)		47 (45.2%)
**Missing**	2	1	1	7	5	16		4
**Diabetes: N (%)**								
**Yes**	4 (11.1%)	7 (19.4%)	6 (16.7%)	5 (13.9%)	11 (22.9%)	33 (17.2%)	**0.673**	17 (15.7%)
**No**	32 (88.9%)	29 (80.6%)	30 (83.3%)	31 (86.1%)	37 (77.1%)	159 (82.8%)		91 (84.3%)
**Vascular-widening procedures (n): N (%)**								
**Yes**	0 (0%)	1 (2.8%)	0 (0%)	2 (5.6%)	3 (6.5%)	6 (3.2%)	**0.359**	1 (0.9%)
**No**	36 (100%)	35 (97.2%)	36 (100%)	34 (94.4%)	43 (93.5%)	184 (96.8%)		107 (99.1%)
**Missing**	0	0	0	0	2	2		0

* p-values: Chi square, Fisher's exact test or analysis of variance

The first 36 consecutive subjects in each group were called up for repeat ABI measurements, and the remaining 14 subjects were registered as stand-bys. This procedure was followed for four groups. Stand-bys underwent only a single ABI measurement (not used for study purposes).

For the ABI repeat measurements, each subject saw exactly one observer from each of the three observer groups (angiologists, GPs and MAs), and each observer measured six of the 36 subjects in a group (Table 2). Each possible two-way combination of angiologist, GP and MA drawn from the six representatives of the three occupations occurred exactly once and was not repeated on a second subject in the same group, as this was a condition for being able to calculate the inter- and intraobserver variability of the ABI measures with only three observers per subject and only six subjects per observer. Thanks to this partially balanced incomplete block design, it was not necessary for all 18 observers to perform two measurements on each of the 4*36 subjects (the usual procedure for duplicate measurements), which would have resulted in a total of 5184 ABI measurements, but only on 4*6 subjects, which resulted in 864 measurements (16.7% of 5184), sufficient to provide the same information with the desired precision.

**Table 2:** Combination schedule of observers and patients

	***Gp1***	***Gp2***	***Gp3***	***Gp4***	***Gp5***	***Gp6***
***AN1***	Pat01 – ***MA1***	Pat02 – ***MA2***	Pat03 – ***MA3***	Pat04 – ***MA4***	Pat05 – ***MA5***	Pat06 – ***MA6***
***An2***	Pat07 – ***MA2***	Pat08 – ***MA3***	Pat09 – ***MA4***	Pat10 – ***MA5***	Pat11 – ***MA6***	Pat12 – ***MA1***
***An3***	Pat13 – ***MA3***	Pat14 – ***MA4***	Pat15 – ***MA5***	Pat16 – ***MA6***	Pat17 – ***MA1***	Pat18 – ***MA2***
***An4***	Pat19 – ***MA4***	Pat20 – ***MA5***	Pat21 – ***MA6***	Pat22 – ***MA1***	Pat23 – ***MA2***	Pat24 – ***MA3***
***An5***	Pat25 – ***MA5***	Pat26 – ***MA6***	Pat27 – ***MA1***	Pat28 – ***MA2***	Pat29 – ***MA3***	Pat30 – ***MA4***
***An6***	Pat31 – ***MA6***	Pat32 – ***MA1***	Pat33 – ***MA2***	Pat34 – ***MA3***	Pat35 – ***MA4***	Pat36 – ***MA5***

AN: Angiologist; GP: General Practitioner; MA: Medical Assistant

Example: Patient 20 (Pat20) is measured by Angiologist 4 (AN4), General Physician 2 (GP2) and Medical Assistant 5 (MA5). Each subject saw exactly one observer from each of the three observer groups, and each observer measured six subjects. Note that this schedule was repeated twice on each patient group of 36 patients to obtain repeat measurements.

### Outcome measures

The primary purpose of the study was the assessment of three sources of variability, namely the true differences in ABI between subjects, the measurement error arising from duplicate measurements of the same subject by the same observer (intraobserver variability), and the additional error arising from measurements by different observers (interobserver variability). Secondary outcome measures were body mass index, waist-hip ratio, and risk factors for atherosclerotic complications (smoking, hypertension, lipid disorders, diabetes mellitus, symptomatic PAD).

### Doppler measurements and ABI determination

The measurements in the study group began after a ten-minute rest period. Each observer was assigned an aide, who was responsible for guiding the observer to the numbered beds in the correct time sequence, as set out in a predetermined list, and for recording the readings on a standard form. The aide ensured the correct sequence of measurements and the blinding of the observer to his/her previous measurements on the same subject. The observers had no access to the measures. Additionally, controllers verified the accuracy of the measurement sequence and procedure at each bed.

Over the course of about 90 minutes, each subject saw three observers, one from each of the occupational groups. Each observer was given a list of six subjects to measure, and always completed all six subjects on the list before returning (approximately 45 minutes later) to the first person on the list to repeat the measurements on the same subjects in the same sequence. The volunteers remained supine between observers, resulting in a resting period of 5–10 minutes between each set of readings (by different observers).

The Doppler ultrasound device with which the ABI readings were taken was the same one used in the getABI study [8,12] (Kranzbühler 8 MHz, Solingen, Germany). Measurements were performed on subjects resting in a supine position with the upper body as flat as possible, since readings taken in the sitting or semi-sitting position may result in a substantial increase in the tibial artery blood pressure. After the initial ten-minute rest, each observer used a blood pressure cuff and a Doppler device to take bilateral readings of systolic blood pressure (SBP) at the anterior and posterior tibial arteries and the brachial artery (in that order). Once the manometer pressure was released, the first pulse sound audible through the Doppler device as the cuff was deflated marked the systolic arterial blood pressure. To eliminate possible noise interference from activity at neighboring beds, headphones were used.

The ratio of ankle SBP to arm SBP yields the ABI value. Calculations were performed according to the recommendations of the American Heart Association [1]. Ankle pressure on either side was the higher of the pressure at either the anterior or posterior tibial arteries on that side. Arm pressure was either the average of the two arm pressure readings, if the difference between the two arms was <10 mmHg, or the higher of the two, if the difference between the two sides was ≥ 10 mmHg. The ABI value for left and right sides was calculated as the left or right ankle pressure divided by the arm pressure. The lower of the two ABI values (right or left) was the subject's overall ABI [13]. Note that on the study day only raw values were recorded. The ABIs were calculated later by the statisticians. All data were double-entered into the data base to ensure accuracy of data entry.

### Sample size

In a previous study [14] the difference between the standard deviations of the most and least experienced observers was found to be 0.05 ABI points (standard deviations of 0.073 and 0.120 respectively). We decided that our study should at least be able to detect a two-way difference between the standard deviations of two observers of half the above value (0.025 ABI points). Given a sample size of 108 (three runs of the design), the power for this comparison in an F-test was calculated as 93%, which is acceptable.

### Statistical analysis

For all statistical tests, two-sided p values <0.05 were considered statistically significant. Characteristics of the ABI study subjects were analyzed using Chi-square or Fisher's exact test for contingency tables, and t-test or one-way ANOVA for the comparison of two or more means.

Each of the ABI values was assumed to represent the sum of the true mean ABI value and random variation from several sources. Analysis of ABI variability was done using a mixed model (Proc Mixed SAS™ Statistical Software Release 9.1.3) with two random factors and their interaction, the subjects (108 levels) and the observers (18 levels). Note that the inclusion of the patient as a factor makes it possible to account for and calculate intra-patient correlation. No repeated measures model was used.

The mean variation of the ABI measures in the case of multiple measurements can be broken down into the following four variance components:

a. Variance of the true ABI values based on mean ABI **between different subjects**, i.e. variance of the ABI that is medically interpreted.

b. Variance of ABI measurements when multiple measurements are performed on the **same subject **by the **same observer **– the so-called **intraobserver variance**, assumed to be due to measurement error and physiological variation.

c. Variance in ABI measurements when multiple measurements are performed on the **same subject **by **different observers **(first component of **interobserver variance**), due to a systematic measurement bias in the individual observer, e.g. an observer generally measuring higher or lower values than others (independent of the specific subject).

d. An additional variance of ABI measurements when multiple measurements are performed on the **same subject **by **different observers**, caused by interaction between subject and observer (second component of **interobserver variance**), i.e. an observer measuring higher or lower values than others *for specific subjects only*. For example, some observers may have a harder time measuring obese subjects than do other observers.

By adding the three variance components of multiple measurements on the **same **subject, namely the **intraobserver variance b **and the two **interobserver variance components c and d**, one obtains the total variance for multiple measurements on the same subject by different observers:

Total variance of all ABI values for a single subject = b + c + d

Simple addition is permissible when the individual variance components are independent of one another. The standard deviation of the ABI measurements for a single subject is the root of the total variance.

In order to evaluate the overall quality of the ABI as a measurement technique for discrimination between different subjects, all four variance components must be considered. An intraclass correlation coefficient (ICC) is normally formed for this purpose. The ICC indicates the proportion (in percent) of the total variance in measurement results between subjects that can be explained by the "biological" or real variance between the subjects examined. A high ICC indicates that the measurements can be used to discriminate between individuals. ICC values ≥ 0.75 are said to be acceptable [15]. The ICC can also be understood as a generalization of the coefficient of determination, or the square of the correlation coefficient in the case of only two sources of variations (two variables). Therefore, the interpretation of the ICC is the same as for the coefficient of determination, namely the proportion of total variance that is explainable by the real variance between subjects.

ICC=aa+b+c+d
 MathType@MTEF@5@5@+=feaafiart1ev1aaatCvAUfKttLearuWrP9MDH5MBPbIqV92AaeXatLxBI9gBaebbnrfifHhDYfgasaacH8akY=wiFfYdH8Gipec8Eeeu0xXdbba9frFj0=OqFfea0dXdd9vqai=hGuQ8kuc9pgc9s8qqaq=dirpe0xb9q8qiLsFr0=vr0=vr0dc8meaabaqaciaacaGaaeqabaqabeGadaaakeaacqqGjbqscqqGdbWqcqqGdbWqcqGH9aqpdaWcaaqaaiabbggaHbqaaiabbggaHjabgUcaRiabbkgaIjabgUcaRiabbogaJjabgUcaRiabbsgaKbaaaaa@3A14@

The intraobserver variability of each individual observer can also be determined. By taking the mean of these values for all observers within one occupational group (angiologists, general physicians and medical office assistants) it is possible to assess the average quality of the measurements by each of the three groups.

Bland-Altman plots were used to visualize measurement errors. For each subject, the differences between the first and second measurements were plotted against the mean of the two measures with 95% limits of agreement [16].

All data analyses were performed using SAS™ Statistical Software (Release 9.1.3, SAS Institute Inc., Cary, NC, USA).

## Results

### Subject characteristics

Of the 200 subjects invited to participate, 192 (96%) appeared on the study day. The data for the pilot study (rehearsal) were not analyzed. Demographic data for all subjects who appeared on the study day are shown in Table 1, subdivided into Groups 1–4 for duplicate ABI determination and a fifth group of stand-bys. The subjects in the five groups were equal for all recorded basic data, including PAD risk factors.

The 108 individuals (53.7% female) for whom complete duplicate measurements were obtained had a mean age of 68.7 (1.5) years, mean BMI of 29.0 (4.3) kg/m^2^, and mean waist-hip ratio of 0.92 (0.09). Active smokers made up 9.3% of the sample and former smokers 40.7%; 58.1% were hypertensive, 54.8% dyslipidemic, and 15.7% diabetic. Only one subject (0.9%) of Groups 1–3 reported having had peripheral vascular-widening measures in the past. In Groups 4 and 5 five subjects (5.9%) reported having had such measures.

### ABI measurements

ABI measurements were performed on 189 of the 192 subjects who enrolled in the study. One subject dropped out after the basic examination, before any ABI determinations were performed, and on two other subjects no ABI measures could be obtained for technical reasons (very obese subjects with upper arm too large for the blood pressure cuff).

In Groups 1–3 (108 subjects), the ABI measurement program was completed as planned with full sets of duplicate measurements. Because of time restrictions, full sets of measurements were not obtained from all subjects in the fourth group. The analysis was based on the first three groups of subjects (58 women, 50 men) for whom complete measurements were obtained.

The results of the ABI measurements are shown in Table 3. Overall 15 (7.9%) subjects had ABI measures <0.90 and would therefore be classified as possibly having PAD. An ABI >1.4, i.e. a ratio that is associated with arterial calcification and increased stiffening of the arterial walls, was not determined in any of the 189 subjects.

**Table 3:** Results of duplicate (Groups 1–4) and single (Group 5) ABI measurements

	**Gr1**	**Gr2**	**Gr3**	**Gr4**	**Gr5**	**Total**	**p-value**	**Gr1-Gr3**
**ABI**								
**- N**	36	36	36	36	45	189		108
**- Mean**	1.10	1.08	1.11	1.06	1.04	1.08	0.055	1.10
**- SD**	0.09	0.11	0.10	0.13	0.17	0.13		0.10
**- Min**	0.94	0.74	0.92	0.6	0.52	0.52		0.74
**- Max**	1.35	1.32	1.37	1.24	1.27	1.37		1.37
**- Missing**	0	0	0	0	0	0		0
**ABI**								
**< 0.90**	0 (0%)	2 (5.6%)	0 (0%)	5 (13.9%)	8 (17.8%)	15 (7.9%)	0.003	2 (1.9%)
**>= 0.90**	36 (100%)	34 (94.4%)	36 (100%)	31 (86.1%)	37 (82.2%)	174 (92.1%)		106 (98.2%)
**Missing**	0	0	0	0	0	0		0

p-values: Fisher's exact test, analysis of variance

There are no significant differences in mean brachial SBP or mean ABI between Groups 1–3 and incomplete Group 4 (Table 4). The mean SBP in Group 5 is not comparable with the mean SBP in the other four groups, because for the stand-by subjects a ten-minute rest period was not scheduled before the single ABI measurement.

**Table 4:** Comparison of mean systolic arm pressure between subjects in stand-by group 5, duplicate measurement groups 1–3, and incomplete group 4

**Brachial SBP**	**Group 1–3**	**Group 4**	**Group 5**	**Total**	**p-value**
**N**	108	36	45	189	
**Mean**	**148**	**148**	**159**	**150**	**0.004**
**SD**	17.0	18.1	26.8	20.4	
**Median**	147	145	158	148	
**25**^**th **^**percentile**	133	136	138	134	
**75**^**th **^**percentile**	158	155	178	161	
**Min**	112	113	110	110	
**Max**	193	199	215	215	
**Missing**	0	0	0	0	

p-values: analysis of variance

### Bland-Altman plots

The simplest and most direct measure of agreement between two measured values is the difference between them. This is true not only when seeking to determine agreement between measures obtained by two different measurement methods, but also when seeking to determine the reproducibility of a measure, in this case an ABI measure, when repeat measurements are performed by the same observer on the same subject.

Bland-Altman plots were created to visualize the ABI measurement error [16]. The difference between two ABI values was plotted against the mean of the same two values, in order to show whether systematic differences exist between the repeat measurements as a function of absolute value (mean) of the ABI. The two blue lines represent two standard deviations of the differences, in other words the upper limit of the difference between two measures up to which about 95% of all the differences between measures can be found. These are the limits of agreement (mean difference ± 2SD) as described by Bland and Altman.

**Figure 2:**
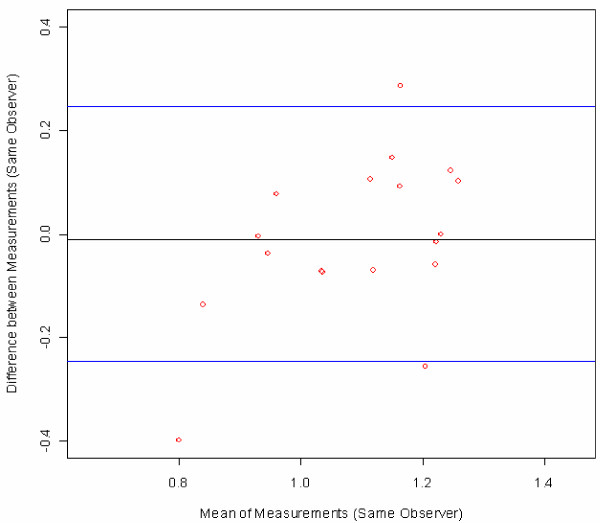
**Bland-Altman plot of all duplicate measurements by one angiologist (Number 4) on a total of 18 subjects in groups 1–3**. Figure legend: red circles = subjects, black line = mean difference, blue lines = ± 2SD; for comparison, the difference between a healthy patient (ABI > 1.1) and a suspected PAD patient (ABI < 0.9) is 0.2 ABI points.

**Figure 3:**
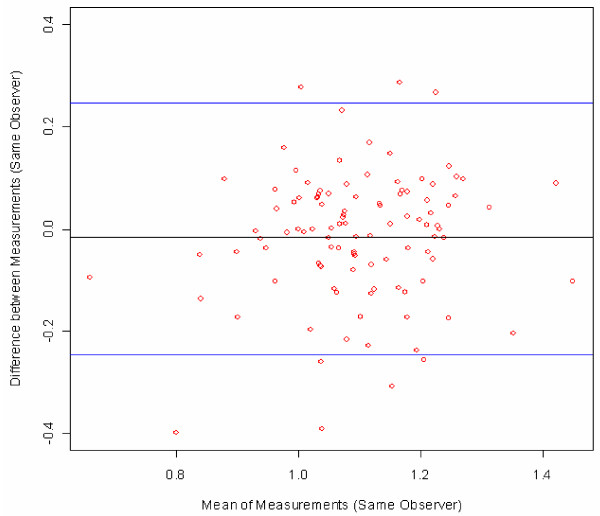
**Bland-Altman plot of all duplicate measurements by all six angiologists on a total of 108 subjects in groups 1–3**. Figure legend: red circles = subjects, black line = mean difference, blue lines = ± 2SD; for comparison, the difference between a healthy patient (ABI > 1.1) and a suspected PAD patient (ABI < 0.9) is 0.2 ABI points.

**Figure 4:**
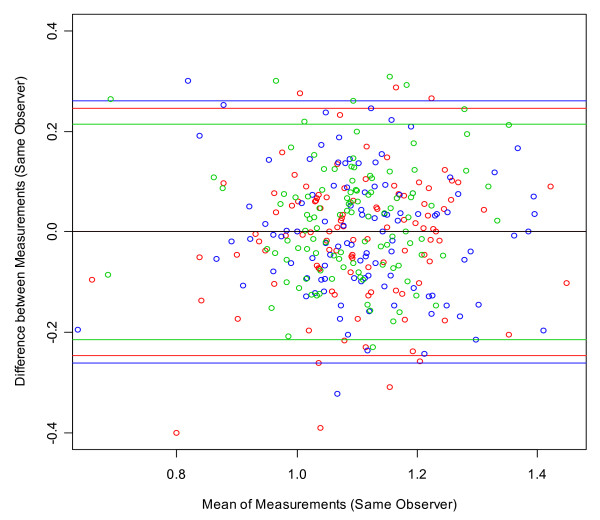
**Bland-Altman plot of all duplicate measurements by all six angiologists (blue), six general physicians (red) and six medical office assistants (green) on a total of 108 subjects in groups 1–3**. Figure legend: red, green and blue circles = subjects; coloured lines = ± 2SD; black line = global mean of differences; for comparison, the difference between a healthy patient (ABI > 1.1) and a suspected PAD patient (ABI < 0.9) is 0.2 ABI points.

**Figure 5:**
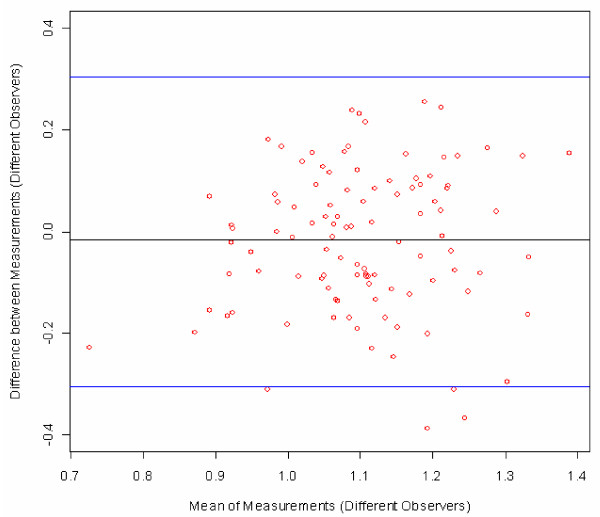
**Bland-Altman plot of difference between first ABI measurements by all six angiologists and six GPs on a total of 108 subjects in groups 1–3**. Figure legend: red circles = subjects, black line = mean difference, blue lines = ± 2SD; in each case, the first measurement by the angiologist is compared to the corresponding first measurement by the GP on the same patient; for comparison, the difference between a healthy patient (ABI > 1.1) and a suspected PAD patient (ABI < 0.9) is 0.2 ABI points.

Figure 2 illustrates the differences between the duplicate ABI measures by a single angiologist (the reproducibility of this observer) for all of his measures (18 subjects, six per group). An analogous plot could be shown for each observer. Figure 3 combines these six plots for all six angiologists in one figure, showing the differences between duplicate ABI measurements obtained by all six angiologists on all 108 subjects in Groups 1–3. Note that each angiologist measured 18 different subjects (6 angiologists * 18 subjects = 108 subjects). Figure 4 combines the differences between duplicate measures on all 108 subjects by all 18 observers (six angiologists, 6 GPs and six MAs). Note that now the plot contains 3*108 points because one subject is measured by three different observers (duplicate measurements by one angiologist, one GP, and one MA). The plotted points in Figure 5 show the differences between all 108 first measurements by 6 angiologists and 108 first measurements by 6 GPs. The mean difference indicates the bias between the two first measurements.

## Discussion

Previously only a few small studies (6–36 subjects) existed on the inter- and intraobserver variability of ABI measures [14,17-19]. These studies worked with small numbers of observers and with patients who had symptomatic PAD or reduced ABIs (<0.95). By contrast, our study focused on a large number of elderly unselected individuals, who are the most likely to be subjects of screening measures, as in primary care. With a total of 18 observers of different technical backgrounds and levels of experience, the study also reflects the everyday conditions of primary care.

From the fact that the mean differences between the ABI measurements in Figure 3 and Figure 5 are very close to 0, the following two conclusions can be drawn: Neither the time difference of about 45 minutes between the two measures on the same subject (repeat measurements by angiologists, GPs or MAs) nor differences in observer category (e.g. angiologists versus GPs) resulted in a systematic bias in the measures. Furthermore, comparison between the two groups of physicians (Figure 5) leads to the conclusion that the ABI measures taken by the GPs are just as accurate as those taken by the angiologists. In sum, Bland-Altman plots show that there are only small differences and no systematic bias between the observers from three occupational groups with different training backgrounds. This confirms the appropriateness of ABI measurements for screening for PAD and generalised atherosclerosis in the GP setting.

Previous studies indicate both that the variability in ABI measures is highly dependent on the experience of the observers, and that the value of the ABI measures depends on the apparatus with which the measurements are performed [14,17-19]. However, the strength of these studies' conclusions must be considered questionable, as all studies had one or more major methodological limitations such as relatively small samples of observers or patients, or the selection of patients with symptomatic PAD. The latter is of importance, as variability has been reported to differ at least slightly between diseased and normal subjects [17,20].

The variability in ABI measures is of particular importance for patients moving from one observer to another, for example as the result of changing family doctors (interobserver variance). Moreover, the ABI should always be an average of several measurements. Under ideal measurement conditions, an ABI <0.9 is considered a readily obtained indicator for the possible presence of PAD [21,22]. The most recent studies nevertheless recommend that patients with ABIs between 0.9 and 1.1 be treated as "borderline PAD", since a 25% higher mortality is found in this group compared to patients with ABIs of between 1.1 and 1.4 [23]. A significant increase in mortality was also recently observed in patients with ABIs >1.4 [24]. Similar results were published by Resnick et al. in 2004; however, because the study population consisted of aboriginal Americans only, the data could not necessarily be applied to the general population [25].

## Conclusion

With the partially balanced incomplete block design, it was possible to test every possible observer combination exactly once while performing only 16.7% of all the repeat ABI measurements that would theoretically be required. Thus we established the conditions for assessing components of variance with large numbers of subjects and observers, which can be applied also to measures other than ABI.

## Abbreviations

PAD: peripheral arterial disease

ABI: ankle-brachial index

BMI: body mass index in kg/m^2^

WHR: waist-to-hip ratio

## Competing interests

The author(s) declare that they have no competing interests.

## Authors' contributions

All authors contributed to the manuscript and gave final approval of the article.

**HGE **is principal investigator, responsible for the conception of the study and the study protocol. He participated in analysis and interpretation of data, and drafted the article.

**CH **was responsible for carrying out the study on the study day, gave logistic support, and drafted the article.

**THL **was responsible for the statistical analysis and interpretation of data, and critically revised the manuscript.

**HJT **obtained the funding, provided statistical expertise and gave final approval of the article.

## Pre-publication history

The pre-publication history for this paper can be accessed here:


